# Online MR evaluation of inter- and intra-fraction uterus motions and bladder volume changes during cervical cancer external beam radiotherapy

**DOI:** 10.1186/s13014-021-01907-1

**Published:** 2021-09-17

**Authors:** Xu Li, Lizhen Wang, Zhen Cui, Yukun Li, Pei Liu, Yungang Wang, Jinhong Zhu, Jianmin Zhu, Yong Yin, Zhenjiang Li

**Affiliations:** Department of Radiation Oncology Physics and Technology, Shandong Cancer Hospital and Institute, Shandong First Medical University and Shandong Academy of Medical Sciences, No.440 Jiyan road, Huaiyin district, Jinan City, 250117 Shandong Province China

**Keywords:** Online MRI, EBRT, Bladder filling, Inter-fractional and Intra-fractional bladder motion

## Abstract

**Purpose/objective(s):**

The purpose of the study was to assess the uterus motions and bladder volume changes of fractional movements in cervical sites throughout the external beam radiotherapy (EBRT) treatment.

**Materials/methods:**

A prospective online MR imaging tracking study was conducted in EBRT 43 patients with at least 4 scans during each treatment (before: ultrasound scan, MRI scan, CBCT scan, after: MRI scan) were included. In order to improve the treatment repeatability, each patient was instructed to empty the bladder and drink 500 ml water 1 h before CT simulation and each treatment. If the ultrasound scan result reached the CT simulation volume of bladder, the treatment began. Bladder was outlined on the T2 weighted axial sequence and CBCT image by the two observers to avoid the influence of contouring. The data of bladder volume and scanning time were accurately recorded. The bladder volumes, filling rates and uterus motion were retrospectively analyzed by MIM software.

**Results:**

Inter-fraction variation of the bladder volume was significant (p < 0.0001). Intra-fraction mean increase of the bladder volume was modest (30 cc) but significant (p < 0.001). Both inter- and intra-fraction of the uterus motion were significant. The average time between the pre-and post-fraction MRI scans was 27.82 ± 7.12 min (range 10–55 min) for IMRT plans and 24.14 ± 5.86 min (range7-38 min) for VMAT plan. Average bladder filling rate was 3.43 ml/min. The bladder filling rate did not change significantly with the course of treatment, but the bladder was more intolerant.

**Conclusion:**

This is the most detailed assessment of intra-fraction and inter-fraction motion during EBRT for cervical cancer. Finally, this study will inform appropriate treatment margins for online adaptive radiotherapy. We suggest that at least one image scan is needed before the EBRT. The portable US scanner provides a quick but unreliable measurement of the bladder volume. There is a significant statistical difference between the results of ultrasonic scanning and that of image scanning.

## Introduction

The EBRT plays an important role in the treatment of locally advanced cervical cancer [1]. During EBRT for cervical cancer, a larger planned target volume was needed to include potential organ movement during treatment. But these huge profits promote the treatment toxicity [2]. The introduction of online adaptive MR-guided EBRT will adapt the plan to the daily anatomical position, but the effect of bladder motion for cervical cancer was not certain [3].

The radiotherapy process of cervical cancer is usually long, the difference of organ movement between different parts has a greater impact on the accuracy of dose delivery [4]. At the same time, population-based standard marginal therapy is ineffective for cervical cancer due to significant differences in the degree and complexity of cervical and uterine movement between patients. Several studies have found that the uterus and cervix can significantly move and disappear during treatment [5–7]. In order to improve the coverage of the target area, the planned target volume around the CTV is to ensure geometric uncertainty during treatment.

Unfortunately, knowledge of what constitutes an appropriate planning margin is currently limited. Although many authors and many methods, such as cone beam computed tomography (CBCT), have extensively studied bladder movement between fractional stages and within fractional levels, there has not been much verification of repeatability and reliability [8–10].

At our clinic, an image-guided cervical radiotherapy protocol is underway that includes pretreatment ultrasound scan as well as intra-fraction positional measurements of the bladder. Pretreatment CBCTs and MRI-scans are acquired at each fraction and include bladder information. But the bladder volume information after treatment is not clear. The purpose of this study is therefore to compare and quantify inter-and intra-fraction bladder volume and uterus position variation during EBRT of cervical cancer by different measured techniques.

## Methods and materials

### Patients and treatment

**Fig. 1:**
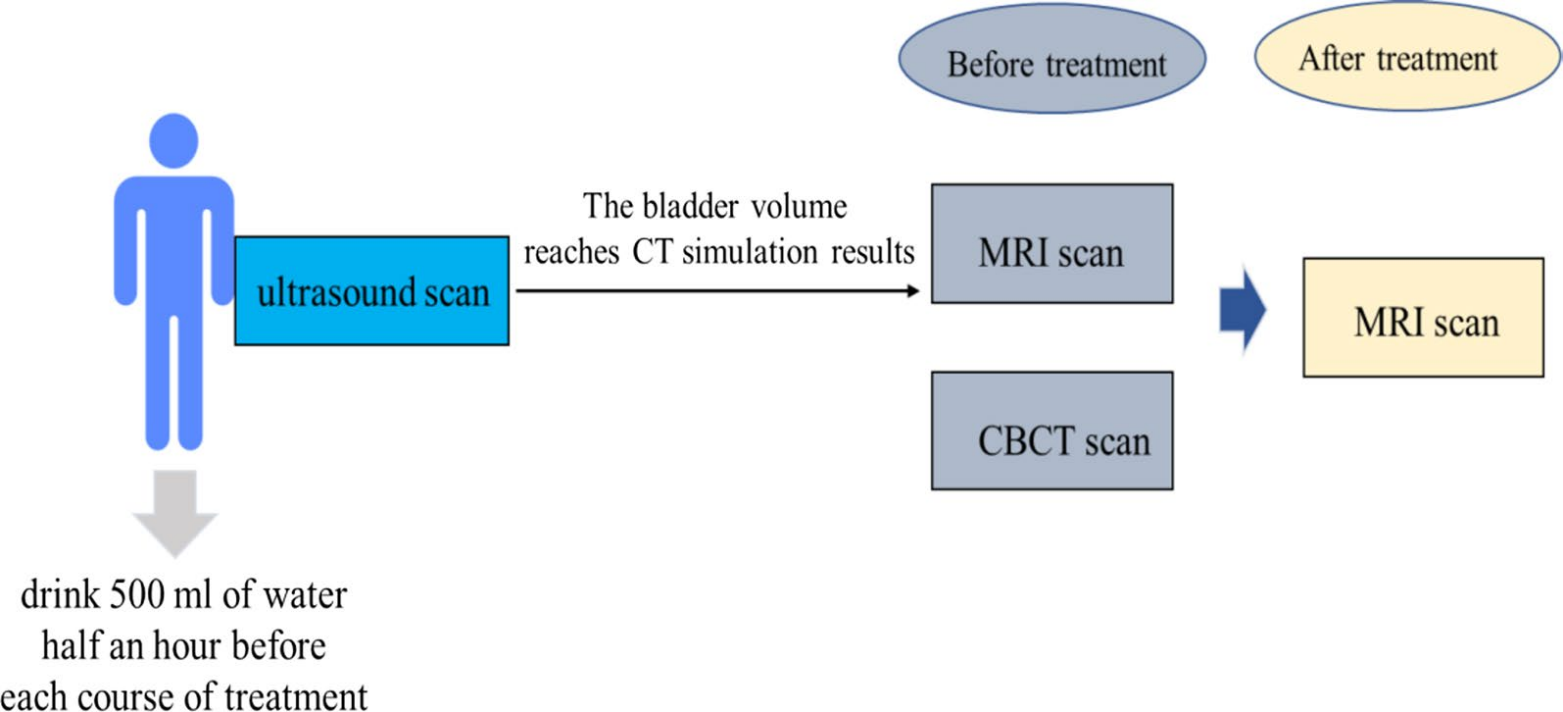
The workflow of scan methods

Forty-three patients with locally advanced cervical cancer patients were prospectively included in this study. The distribution of FIGO (International Federation of Gynaecology and Obstetrics) stages was as follows: IB: 3 patients, IIA: 3 patients, IIB: 6 patients, IIIA: 12 patients, IIIB: 15 patients, and IV:4 patients. The patients’ ages ranged from 26 to 67 years (median 49). Radiotherapy combined external beam radiotherapy (EBRT) with brachytherapy delivered in the last 1–2 weeks of EBRT [11]. For 13 patients, radiotherapy was combined with either chemotherapy. The doses at the ICRU reference point were 46 or 50 Gy, which were delivered in 23 or 25 daily fractions of 2 Gy (4–5 weeks of full treatment course). In an attempt to achieve day-to-day reproducibility in bladder filling, the patients were asked to drink 500 ml of water 1 h prior to each EBRT treatment fraction (Fig. 1). Patients were treated in supine position on a belly board and with a foot rest.

### Data acquisition and delineation

In each session of each patient, five scans were obtained at different points in time. The first image scan was CT simulation. As for the simulation, patients are required to drink 500 ml of water an hour before simulation. In order to achieve consistent results, before CT simulation acquisition, the bladder volume was measured using a portable bladder US scanner. Simulation scan starts only when the volume of the bladder reaches CT simulation standard, and the corresponding volume measurement is carried out by portable US scanner. As for daily treatment, patients were also asked to drink 500 ml of water 1 h prior to each session. The volume of bladder was detected by portable US scanner before each treatment. When the volume of bladder reaches CT simulation standard, the treatment process started. MRI scan is performed when the urine volume meets the requirements (the same bladder volume as CT simulation), which was scanned by ultrasound. This study uses fast pelvic MRI sequence, the scan time is 2 min. The scanning parameters of Unity are ACQ matrix = 268 × 267, FOV = 400 × 400 mm, TR = 1532 ms, TE = 278 ms. After acquisition of this MRI scan, the patient is asked to do radiotherapy. Before radiotherapy, the CBCT-scan was performed. After the radiotherapy, the MRI-scan were did again by using the same MRI sequence. The bladder wall was delineated by two experienced radiographers on planning CT-scan and each pre- CBCT-scan, MRI-scan and post MRI-scan by using Monaco 5.40.01 version treatment planning system (TPS).

### Analysis of bladder volume and position change

Bladder volume change rate and uterus motion were analyzed by separately. Bladder volumes were calculated by the manual delineation data. The volume increase was calculated by CBCT scan and MRI scans, and the pre-treatment bladder volume was subtracted from the post-treatment volume. The filling rate was then calculated by dividing the volume increase by the time between pre-treatment scans and post-scan MRI scan recorded by the device. Bladder volume change rate and uterus motion were analyzed by using MIM software (the version was 7.0.4).

### Statistics

The statistics of bladder volumes and uterus motion was based on CBCT-scan bladders, MRI-scan bladders, and the planning CT bladders. Statistics of the variations for the population of patients was calculated as follows: The group mean value was calculated as the average value of each patient, and the systematic error (P) was the standard deviation (SD) of the average value per patient. The root mean square error (r) of all SD patients in all scores of each patient was calculated. We tested whether group statistics significantly differed between the different scan groups. Mean inter-fraction bladder movement was compared using an independent-samples t-test.

## Results

**Fig. 2:**
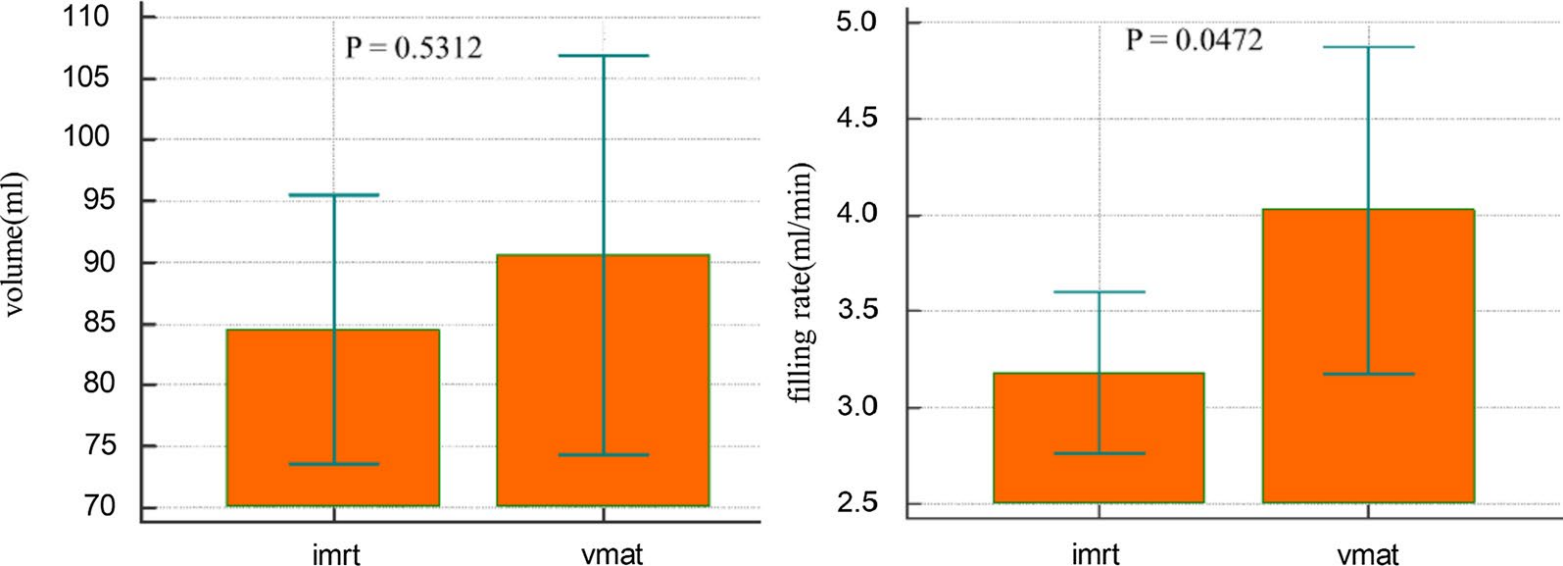
There is no statistical difference (p = 0.5312) in bladder volume pre- and post treatment in imrt and vmat treat mode. The bladder filling rate is statistically different between imrt and vmat (p = 0.0472)

The mean time between the pre-and post-fraction MRI scans was 27.82 ± 7.12 min(range 10–55 min) for IMRT plans and 24.14 ± 5.86 min (range7-38 min) for VMAT plan. A summary of the results can be found in Table 1. As the Fig. 2 showed that there is no statistical difference (p = 0.5312) in bladder volume pre- and post-treatment in imrt and vmat treat mode. The bladder filling rate is statistically different between imrt and vmat(p = 0.0472). A total of 215 CBCT-scans, 476 MRI-scans, 281 Ultrasound-scan and 43 planning CT-scans were analyzed. All patients were able to follow all treatment flow. The bladders were delineated in all scans except ultrasound scans.

**Table 1:** Variations in bladder volume and filling rate

	IMRT		VMAT	
	Pre-treatment	Post-treatment	Pre-treatment	Post-treatment
	Mean		Mean	
Volume (ml)	335.16	419.67	268.39	359.03
Vpre – Vpost (ml)	84.51		90.63	
dV/dt (ml/min)	3.18		4.03

### Inter-fraction volume and position variation

**Fig. 3:**
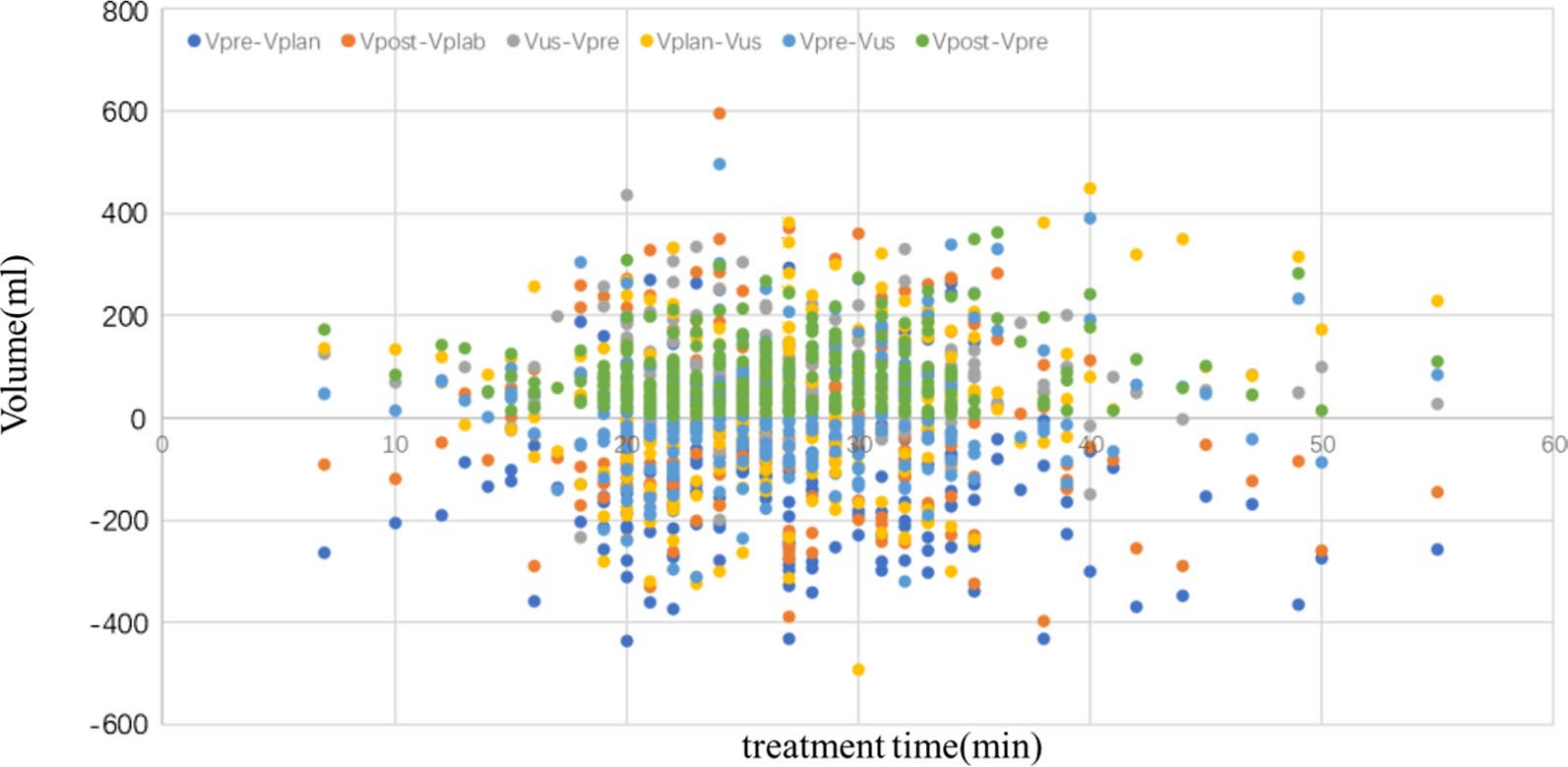
Distribution of bladder volume with treatment time. The Vus: the bladder volume of US scanner measured; Vpre: the bladder volume of MRI scanner measured before treatment; Vpost: the bladder volume of MRI scanner measured after teatment. Vplan: the bladder volume of CT simulation scan

**Fig. 4:**
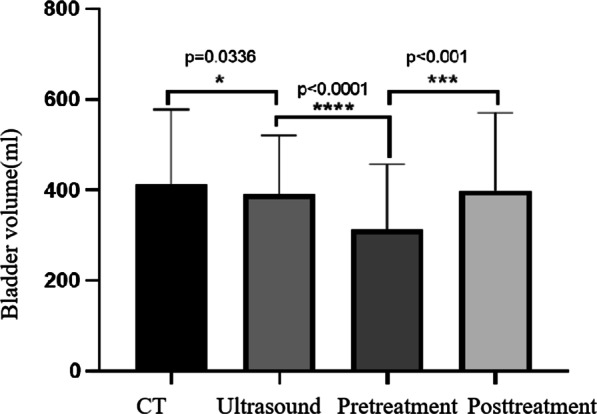
The distribution of bladder volume under different measurements.There was no significant statistical difference between ultrasound scan and the post treatment scan.One asterisk mean that the p value was less than 0.01.Three asterisks mean that the p-value was less than 0.001.Four asterisks mean that the p-value was less than 0.0001

A summary of the results can be found in Table 2 and Fig. 3. Bladder filling rate was 3.43 ± 3.05 ml/min over all treatment fractions. A significant positive correlation was found between bladder filling rate and volume of the bladder at the start of a fraction, as measured in the pre-fraction MRI scan (r = 0.46, p < 0.01). This means that a larger bladder volume observed at the start of treatment was predictive of a higher inflow rate. Regression analysis showed a constant of 1.5 and a coefficient of 0.007, which means that for every 100 ml extra bladder volume at the start of treatment, the inflow rate increased with approximately 0.7 ml/min. As shown in Fig. 4, the bladder volume in plan CT is statistically different from the volume measured by ultrasound (p = 0.0336), and the volume measured by ultrasound is statistically different from the volume before treatment (p < 0.0001), but there is no statistical difference between the ultrasound results and after treatment(p = 0.2595).

**Table 2:** Variations in bladder volume for all patients. The group x means number of treatments (x − 1) * 5 + 1 ~ (x − 1) * 5 + 5

	All patients		Group1 (1–5)		Group2 (6–10)		Group3 (11–15)		Group4 (16–20)		Group5 (21–25)		Group6 (26–30)	
	Mean	σ	Mean	σ	Mean	σ	Mean	σ	Mean	σ	Mean	σ	Mean	σ
Treat time (ml)	26.84	6.94	27.65	8.20	27.18	7.29	27.00	6.87	26.34	5.59	23.96	4.76	28.69	5.72
Vpre − Vplan (ml)	− 96.88	143.46	− 97.98	169.11	− 92.52	139.66	− 75.33	141.14	− 99.89	99.52	− 112.27	155.17	− 140.72	112.66
Vpost – Vplan (ml)	− 10.52	152.88	− 2.64	176.17	8.02	154.22	13.94	146.09	− 24.87	113.13	− 46.57	154.85	− 81.56	125.96
Vus – Vpre (ml)	77.25	86.76	61.29	106.78	75.77	81.26	84.24	81.87	75.37	59.81	98.62	87.79	98.15	69.24
Vplan – Vus (ml)	19.63	148.89	36.69	160.11	16.74	145.36	− 8.91	158.96	24.52	98.93	13.65	178.22	42.56	126.19
Vpre – Vus (ml)	9.11	116.47	34.05	140.01	24.76	105.70	5.02	123.70	− 0.34	88.27	− 32.92	93.74	− 39.00	77.48
Vpost – Vpre (ml)	86.37	70.63	95.34	83.17	100.54	65.21	89.27	81.27	75.02	58.02	65.70	39.35	59.15	50.60
(Vpost − Vpre)/dt	3.43	3.05	3.68	3.33	4.26	4.03	2.34	1.76	2.93	2.16	2.90	1.92	2.33	2.30

Bladder volume increased on average by 86.37 ± 70.63 over all treatment fractions. The median bladder volume increase rate of the whole group was 3.43 ml/min, with an intra-patient SD of 3.05 ml/min. As the treatment progresses, the rate of bladder volume change gradually decreases(from 3.68 ± 3.33 to 2.33 ± 2.30 ml/min, p < 0.05). The Vus(the volume of US scanner measured) is statistically different from the Vpre (the volume of MRI scanner measured before treatment)and Vpost (the volume of MRI scanner measured after teatment).

The maximum mean directional displacements of the bladder walls for the 43 patients over the scan for the full bladders were 10.8 mm superiorly, 1.5 mm inferiorly, 3.19 mm anteriorly, 3.43 mm posteriorly, 2.74 mm to the left and 2.48 mm to the right. Largest displacements are found in the cranial-anterior direction. Average displacement per treatment fraction was 3.6 mm in that direction. In 15.6% of the cases, we found a dis-placement equal or more than 14 mm in the cranial-anterior direction. In the AP and CC direction the group-mean was significantly different from 0 (p < 0.05), indicating a preferred direction for the systematic intra-fraction displacements. The movement of the bladder during treatment and the resulting cervical-uterine movement seems to be a reasonable explanation. A similar trend was observed in the AP and CC direction.

## Discussion

Accurately accounting for daily uncertainties in target position is an important goal in treating cervical cancer [12]. Uncertainties can arise due to setup variation and both inter- and intra-fraction motion [13]. While increasingly rapid treatment times may mitigate the effects of intrafraction motion, inter-fraction motion is generally addressed by encompassing the target with a planning margin[14]. Presently, quantitative evidence supporting recommended planning margins for intact cervical cancer remains sparse, and the effects of inter-fraction changes on CTV coverage are not fully understood [15–17].

It has been shown that the variation in initial bladder volume was large mean SD 70.63 cc, despite the fact that consistent drinking and voiding instructions were followed prior to acquisition. This could be due to several effects. First, we found that some patients (No. 4, 7,18,26, and 33) had difficulties completing the whole treatment process. Second, the waiting time before the start of a treatment was not always equal, and this could the main reason in variation in initial volume.

This study is, to our knowledge, the first to use daily pre- and post-fraction MRI scans to quantify changers in bladder and uterus motions. The results showed that inter-fraction and intra-fraction cervix-uterus motion were considerable in each treatment. The average intra-fraction (inter-fraction) displacement of the group was 6.11 mm (5.9 mm) in the AP direction and 11.5 mm (10.3 mm) in the CC direction. As shown in Fig. 4, there is a certain difference between the volume measured by ultrasound and the volume measured by planned CT. We currently lack effective means to ensure the consistency of bladder volume. At the same time, it can also be seen that the bladder volume changes significantly with the treatment process.

Individual patient displacements may be even larger and extend more than 10 mm. In this study, we also observed that there was a significant correlation between bladder volume difference and cervical-uterine displacement (r = 0.61, p < 0.01). Obviously, variations in the position and shape of the bladder were primarily caused by changes in bladder filling. As many studies have shown that MR-guided daily-adaptive radiotherapy seems a promising approach for minimizing treatment uncertainties and to improve treatment outcome. Nicosia et al.[18] found MR-guided daily-adaptive SBRT seems a feasible and accurate strategy for treating prostate cancer with ablative doses. The MR-guided strategy provides a comparable level of accuracy and acceptable real-dose distribution over treatment fractions. Francesco et al. [19] using 1.5 T MR-guided radiotherapy to assess daily variations for both target coverage and organs-at-risk. The study found that daily-adaptive can improve single intestinal loop sparing for lymph-nodal oligometastases. The same team also study the organ motion is a crucial feature for prostate SBRT. They shown that MRI-Linac is a new frontier in radiation oncology in showing visualization of the real-time anatomy of the patient[20, 21].

As other investigators have found [10, 22], we observed significant motions in bladder volume and uterus. Agreements to ensure that these organs are consistent in size will help reduce the source of CTV location changes. For interfraction motion, Yee et al. [23] found in a landmark study of bladder cancer CBCT that the movement is the most significant in anteriorly. Without daily imaging guidance, a large part of the target volume may be lost. Haripotepornkul et al.[24] using cervical implanted markers, daily kV imaging was used to study the intra-segmental movement of patients with cervical cancer throughout the IMRT process. An intermediate marker offset of 2–4 mm is observed in any given direction, which is smaller than our analysis, especially in the AP direction. However, this can be explained because the markers only represent the position of the cervix, and the cervix is affected by changes in bladder filling smaller than the uterus motion [8].Chen et al. found that the largest movement was found at the bottom of the uterus over a time range of 31 min, averaging 1.1 mm in the AP direction and 3.1 mm in the CC direction [25]. This is smaller than what we found, however, it can be explained that we only include patients with large uterine tip displacement measured in full and empty bladder CT scans before treatment [26]. In addition, we would like to point out that we analyzed the scans in pairs, that is, we compared the pre-and post- MRI and CBCT scans of each part. In this way, the effect is mainly reduced to the interpretation difference between pre- and post- image scans. In addition, the accuracy of non-rigid registration used to measure intra-graded motion may be a potential source of error [27]. However, we have seen that this error is very small, so its effect on the observed internal motion is limited. In this study, we used pre-and post-fractionated CBCT scans to quantify the shape of cervix and uterus, and bladder filling, and intra-fractionated changes in patient settings. There is almost no change in the amount of exercise set by the patient. The movement of cervix and uterus is quite large, which is related to bladder filling [28]. The gradual cervical-uterine movement should not be ignored, especially when designing daily planning strategies. The maximum mean directional displacements of the bladder walls over the 43 patients over the scan for the full bladders were 10.8 mm superiorly, 1.5 mm inferiorly, 3.19 mm anteriorly, 3.43 mm posteriorly, 2.74 mm to the left and 2.48 mm to the right. Largest displacements are found in the cranial-anterior direction. Average displacement per treatment fraction was 3.6 mm in that direction. Statistically significant differences were seen in the posterior, left and right displacements but were quantitatively small.

Patient factors may lead to inter-and intra-temporal changes in the size and position of the bladder. Cervical cancer, especially in patients receiving radiation therapy, usually occurs in the elderly. Despite formal recommendations, deterioration in bladder and renal function may limit a patient's ability to continuously empty the bladder and may also affect the rate at which urine fills the bladder [29]. Adamson and Wu[30], in a study of pretreatment and posttreatment CBCT in prostate cancer patients, found that the median bladder increase was 14%. As expected, we found that the largest proportion of bladder volume increase before and after treatment was in patients with small bladder before treatment. This may simply reflect a relatively constant rate of urine production; therefore, patients with a smaller bladder before treatment have a relatively larger increase in volume than patients with a larger bladder before treatment. This suggests that a larger bladder may require a smaller edge than a smaller preconditioned bladder if the CTV-PTV edge is personalized to take into account intra-segmented movement. Although this hypothesis is logical, it needs to be confirmed by further research.

## Data Availability

Not applicable.

## Abbreviations

EBRT: External beam radiotherapy treatment
CBCT: Cone beam computed tomography
CTV: Clinical target volume
FIGO: International federation of gynaecology and obstetrics
US: Ultrasound
FOV: Filed of view
TR: Repetition time
TE: Echo time
TPS: Treatment planning system
SD: Standard deviation
