# Correction to: Online MR evaluation of inter- and intra-fraction uterus motions and bladder volume changes during cervical cancer external beam radiotherapy

**DOI:** 10.1186/s13014-021-01922-2

**Published:** 2021-10-12

**Authors:** Xu Li, Lizhen Wang, Zhen Cui, Yukun Li, Pei Liu, Yungang Wang, Jinhong Zhu, Jianmin Zhu, Yong Yin, Zhenjiang Li

**Affiliations:** Department of Radiation Oncology Physics and Technology, Shandong Cancer Hospital and Institute, Shandong First Medical University and Shandong Academy of Medical Sciences, No.440 Jiyan road, Huaiyin district, Jinan City, 250117 Shandong Province China

## Correction to: Radiation Oncology (2021) 16:179 10.1186/s13014-021-01907-1

After publication of this article [1], the authors reported that Jinhong Zhu was incorrectly denoted as one of the corresponding authors during the publication process. The publisher apologizes for the inconvenience caused to the authors and readers.

The original article [1] has been updated.
